# Effect of Nrf2 on brain injury induced by hydraulic shock via regulation of mitophagy and apoptosis

**DOI:** 10.18632/aging.205250

**Published:** 2023-11-28

**Authors:** Erwei Zhang, Tongmao Wu, Yayu Zhuo, Junling Cui, Si Sun, Guobiao Wu, Gengshen Zhang

**Affiliations:** 1 The Second Hospital of Hebei Medical University Department of Neurosurgery, Shijiazhuang, China

**Keywords:** Nrf2, mitochondrial autophagy, apoptosis, hydraulic shock brain injury

## Abstract

The specific protective mechanism of mitophagy and Nrf2 in brain injury has not been fully clarified. This study aimed to reveal the effect of Nrf2 on hydraulic shock brain injury in mice, and explore its possible mechanism. Twenty-four Nrf2 knockout (Nrf2-/-) and wild-type mice (WT) of C57BL/6J were randomly divided into two groups: control group (C) and brain injury group (TBI). Hematoxylin-eosin staining (HE) assay was used for the histomorphological observation. The apoptotic state of brain tissue was detected by TUNEL. Mechanical damage *in vitro* models of glial cells were prepared. The wild-type (WT) and Nrf2 knockout (KO) mice were constructed to investigate the changes of mitophagy and apoptosis-related indicators by Western blotting. The experimental results showed that 24 h after TBI, the tissue structure was highly porous, the cells were highly edema, the neuronal space increased significantly, the neuron degeneration, and the cell vacuolation was obvious. Meanwhile, the number of apoptotic cells and the apoptosis rate of glial cells increased significantly. After injury, the relative expression of Parkin, Pink, Beclin and LC-3II proteins were significantly decreased in all mice. The protein expressions of Caspase3 and Caspase12 were significantly increased. However, in the TBI group, KO mice were more impaired than WT mice. In conclusion, Nrf2 plays a protective role by promoting mitophagy to inhibit apoptosis in the process of brain injury caused by hydraulic shock in mice, which provides a new idea for the effective treatment of brain injury.

## INTRODUCTION

Traumatic brain injury (TBI) is one of the important diseases that seriously threaten human health, especially the young and middle-aged people. Ischemia and hypoxia induced by traumatic brain injury initiate a series of pathological biochemical processes, including the destruction of the blood-brain barrier, oxidative stress, inflammation and apoptosis [1, 2]. Apoptosis of nerve cells plays an important role in secondary craniocerebral injury [3]. Therefore, inhibition of nerve cell apoptosis can improve the nerve damage after TBI.

Autophagy and apoptosis are mutually influenced. Autophagy inhibited apoptosis when the environmental condition was less affected. However, when autophagy depletes the proteins and organelles in the cell, the cell can no longer survive, and the cell converts to apoptosis [4]. The main mechanism for autophagy to inhibit apoptosis is mitophagy, which is a complex process involving the interaction between mitochondria and autophagy [5, 6]. Selective sorting and removal of damaged or unwanted mitochondria and preventing these organelles from initiating the apoptotic process of mitochondrial pathways are key mechanisms for maintaining mitochondrial function [4]. Mitophagy plays an important protective role in the pathogenesis of nervous system diseases and its activation is involved in important proteins [7]. LC3 is the most common marker of autophagosome membrane, and its content often reflects the degree of autophagy. Beclin-1 is a positive regulatory protein of autophagy [8]. Parkin is a mitochondrial serine/threonine kinase on the outer membrane of mitochondria that prevents mitochondrial apoptosis from PTP opening and cytochrome C release [9]. Pink1 plays an upstream role in the gene pathway that regulates mitochondrial phagocytosis [10]. Moreover, mitosis has been shown to be highly regulated by the Pink1/Parkin pathway, a classical mitotic signaling pathway [11]. The loss of Pink1 results in defective mitophagy [12].

In recent years, more and more studies have shown that Nrf2 plays a brain protective role in TBI [7, 13]. Based on the important role of Nrf2-related signaling pathway in anti-oxidative stress regulation of autophagy [14]. Nrf2 may be a target for the treatment of traumatic brain injury. Nrf2 has been shown to be an emerging gene regulating cell expression, encoding antioxidant enzymes, detoxification factors, anti-apoptotic proteins and drug transporters, which can regulate the gene expression of a variety of cell protective proteins [15, 16]. In addition, new evidence suggests that Nrf2 regulates mitochondrial function and metabolism [17]. Alleviating oxidative stress and mitochondrial dysfunction through Pink1-mediated mitochondrial phagocytosis [7]. However, the specific protective mechanism of Nrf2 in brain injury is not fully understood.

Therefore, in this study, glial cells were cultured *in vitro* and mechanical pressure was applied to simulate traumatic brain injury. The changes of autophagy-related proteins and cell apoptosis after TBI were observed, and the specific mechanism of Nrf2-mediated mitophagy in improving brain injury by hydraulic shock was explored. At the same time, the effects of Nrf2 blockade on autophagy and apoptosis were observed by constructing wild-type and Nrf2 knockout mice models of brain injury by hydraulic shock, so as to provide a new idea for the effective treatment of brain injury.

## MATERIALS AND METHODS

### Access to public data

From the GEO (Gene Expression Omnibus) database https://www.ncbi.nlm.nih.gov/gds/ GSE24265 cerebral hemorrhage related data sets were searched and downloaded. R language limma software package was used for GSE24265 data sets quantile standardized preprocessing, and differences in gene analysis (|logFC|<0.5, *p*-value<0.05), and to get the related differentially expressed genes, and the volcano map and cluster analysis heat map for the differentially expressed genes were drawn.

### Functional enrichment analysis

GO (Gene Ontology) enrichment analysis and KEGG (Kyoto Encyclopedia of Genes and Genomes) enrichment analysis were performed on the common differentially expressed genes obtained from GSE24265 dataset. DAVID (https://david.ncifcrf.gov) online tools were used to GO GSE24265 mRNA differences between genes and KEGG enrichment analysis. GOPlot and ggplot2 package were used to plot the GO pathway and KEGG pathway enrichment analysis of differentially expressed genes in R language environment.

### Gene set enrichment analysis

Gene Set Enrichment Analysis (GSEA) tool (http://www.gsea-msigdb.org/) was used for GSEA enrichment analysis of all genes, and GSEA enrichment analysis pathway map was drawn.

### Statistical analysis of target genes

Two independent samples t-test were used to compare the two groups of data. GraphPad Prism software was used for drawing. *P* < 0.05 was considered statistically significant.

### Preparation of animal models

Twenty-four Nrf2 knockout (Nrf2-/-) and wild-type mice (WT) of the 5-week-old male C57BL/6J strain were purchased from the Second Hospital of Hebei Medical University. They were placed in a suitable environment (20±2° C, 55±5% relative humidity, and 12/12 h light/dark cycle). They were fed with standard diet and distilled water. KO mice and WT mice were randomly divided into two groups: control group (C) and brain injury group (TBI), with 6 mice in each group (including WT mice, Nrf2-/- mice, WT + fluid percussion brain injury mice, and Nrf2-/- + fluid percussion brain injury mice). This study was approved by the Laboratory Animal Welfare and Ethics Committee of Hebei Medical University, and all protocols followed the ARRIVE (2.0 version) guideline.

Mice in the TBI group received intraperitoneal injection of 2% pentobarbital (100 mg/kg, purchased from Sigma Company, USA), then the skin of the head was prepared and disinfected with iodine volt. The skin and subcutaneous tissue were cut along the median longitudinal, and the periosteum of the skull was cut, 0.4 cm beside the right sagittal suture and 0.4 cm behind the coronal suture as the center point. The bone window with a diameter of about 0.3 cm was drilled with an electric grinding-drill to keep the dura intact and stop the bleeding with bone wax. Mouse head fixed in the fixed frame and adjusted to the appropriate location and height, bone window connection shock tube, denture acrylic bone window close and shock tube connectors, to set the equipment for the hydraulic impact of brain damage strength to 0.05 MPa, trigger switch, high pressure gas drive reservoir tank clean saline impact mice dura mater, completed a potential damage model. Mice were removed, gentamicin saline was used to flush the operative area, bone wax was used to seal the bone window, and scalp incisions were closed. Mice in group C were not treated.

### Specimen collection and production

After 24 h of hydraulic shock, the mice were killed by decapitation, and their brains extracted from the ice plate. About 100 mg of brain tissue was taken from the front edge of the injured area, and the water content of brain tissue was measured by dry/wet weight method. The brain tissue at the posterior edge of the injured area was quickly placed in liquid nitrogen, and then rapidly frozen in liquid nitrogen, it was placed in a refrigerator at -80° C for long-term preservation for subsequent experiments.

### Hematoxylin-eosin staining (HE)

After the damaged area was cut into coronal slices of about 3 mm and fixed in 4% paraformaldehyde for 24-36 h, dehydration, transparency, paraffin impregnation and embedding were performed according to the conventional procedures. After paraffin was completely dried and fixed, tissue sections were made with a thickness of 4 μm. Brain tissue was stained according to the instructions of HE staining kit. The brain tissue was dewaxed to water, stained with hematoxylin solution for several minutes, rinsed with tap water, divided with 1% hydrochloric acid alcohol for several seconds, rinsed with tap water, then returned to blue with 1% ammonia solution for 1min, rinsed with running water for several seconds, put into eosin staining solution for several seconds, rinsed with running water. Paraffin sections were put into 75% ethanol for 2 min, 85% ethanol for 2 min, anhydrous ethanol for 5 min, anhydrous ethanol for 5 min, xylene for 5 min, and then the sections were sealed with neutral gum from xylene. And the morphological and structural changes of mouse brain tissue were observed under light microscope.

### *In situ* end labeling (TUNEL) analysis

TUNEL assay was used to detect the apoptotic state of brain tissue. Paraffin sections were dewaxed to water according to the conventional method, 1% Triton X-100 at room temperature and washed 3 times with PBS after permeabilization. The tissue sections were covered with protease K working solution and incubated in an incubator at 37° C for 30 min. 50 μL of TdT enzyme reaction solution dropwise was added, and put into a wet box, and react at 37° C for 60 min, avoiding light, and washed with PBS. 50 μL Streptavidin-TRITC labeling solution dropwise was added into a wet box, reacted for 30 min at 37° C, avoiding light, and washed with PBS. The nuclei were stained with 4,6-diamidino-2-phenylindole (DAPI) and washed with PBS, and observed under fluorescence microscope. The ratio of apoptosis was the number of apoptotic neurons/total number of neurons.

### Western blot

RIPA lysate and phenylmethane sulfonyl fluoride (PMSF) were used to extract mouse brain tissue protein samples. Protease inhibitor PMSF was added according to PMSF:RIPA = 1:100 and homogenized on ice. The supernatant was centrifuged at 12000 r at 4° C for 15 min. The supernatant was divided into 200 μL EP tubes and stored at -20° C. BCA kit was used to detect the protein concentration in the supernatant. The loading volume of the sample was calculated according to the protein concentration and loading system of the sample. Ultrapure water and 5× loading buffer were added, and the metal bath was denatured at 100° C for 10 min after mixing and centrifugation. The protein samples were subjected to SDS-PAGE in 5% concentrate gel and 10% separation gel, and the target protein was transferred to PVDF membrane by membrane transfer. Then 5% skim milk powder was sealed at room temperature for 2 hours and incubated overnight with primary antibody at 4° C. After the primary antibody was incubated overnight, the PBST membrane was washed three times for 10 min each time. The membrane was incubated in horseradish peroxidase labeled secondary antibodies at room temperature for 2 h, and then washed 3 times with PBST. Finally, the exposure was performed with the ultra-sensitive ECL chemiluminescence kit and Fluorchem Q instrument, and the protein quantitative analysis was performed with the AlphaView software system.

### Extraction and culture of glial cells

Newborn wild type (WT) and Nrf2 gene knockout (KO) suturing mice were killed within 24 hours and soaked in 75% ethanol for disinfection. The meninges and blood vessels were removed. The brain (hippocampus part was removed) was placed in PBS, and the surface blood filaments were rinsed and removed until the brain tissue was milky white. Tissue was torn into chyme with ophthalmic tweezers, and then repeatedly cut with ophthalmic scissors for 10 min. Finally, the tissue was gently blown with pipette gun for 20-30 times. The torn tissue was transferred into a 15 mL centrifuge tube with the gun and digested in a water bath at 37° C for 15 min. Digestion stop solution was added to stop the enzyme reaction. Centrifuged at 1000 rpm for 10 min, supernatant was discarded, culture medium was added to resuspend, and cells were stained and counted. Cells were inoculated at a density of 1×10^6^ cells/mL into culture flask and placed in an incubator at 37° C with 5% CO_2_. The appropriate size of the glass slide disinfection treatment was selected, a small amount of culture medium was added to make it adsorbed on the bottom of the six-hole plate. Cells were inoculated and cultured for 24 h, with a density of about 8000 cells per well, and treated in different groups: control group (group C) and brain injury group (TBI group, mechanical pressure was applied to simulate traumatic brain injury).

### Preparation of mechanical damage models *in vitro*

According to Balentine et al., this kind of experimental model was firstly developed to prepare *in vitro* mechanical compression injury model [18]. A 25-105 mg tailor's needle is dropped directly from a 10 cm high plane onto the exposed surface of the culture, causing the seemingly mature normal culture to suffer temporary shock trauma. Follow-up experiments were performed at a selected post-traumatic time interval (24 h).

### Statistical analysis

All data were expressed as mean ± standard deviation $\left(\bar{\text{x}}\pm \text{s}\right)$, and SPSS 22.0 statistical software (IBM, USA) was used to conduct Student’s t test for the differences in all indicators between groups. Analysis of variance (ANOVA) was adopted for multivariate analysis and SNK was used for post hoc test of differences in means. *P*<0.05 indicates that the difference is statistically significant.

## RESULTS

### Histomorphological observation

**Figure 1:**
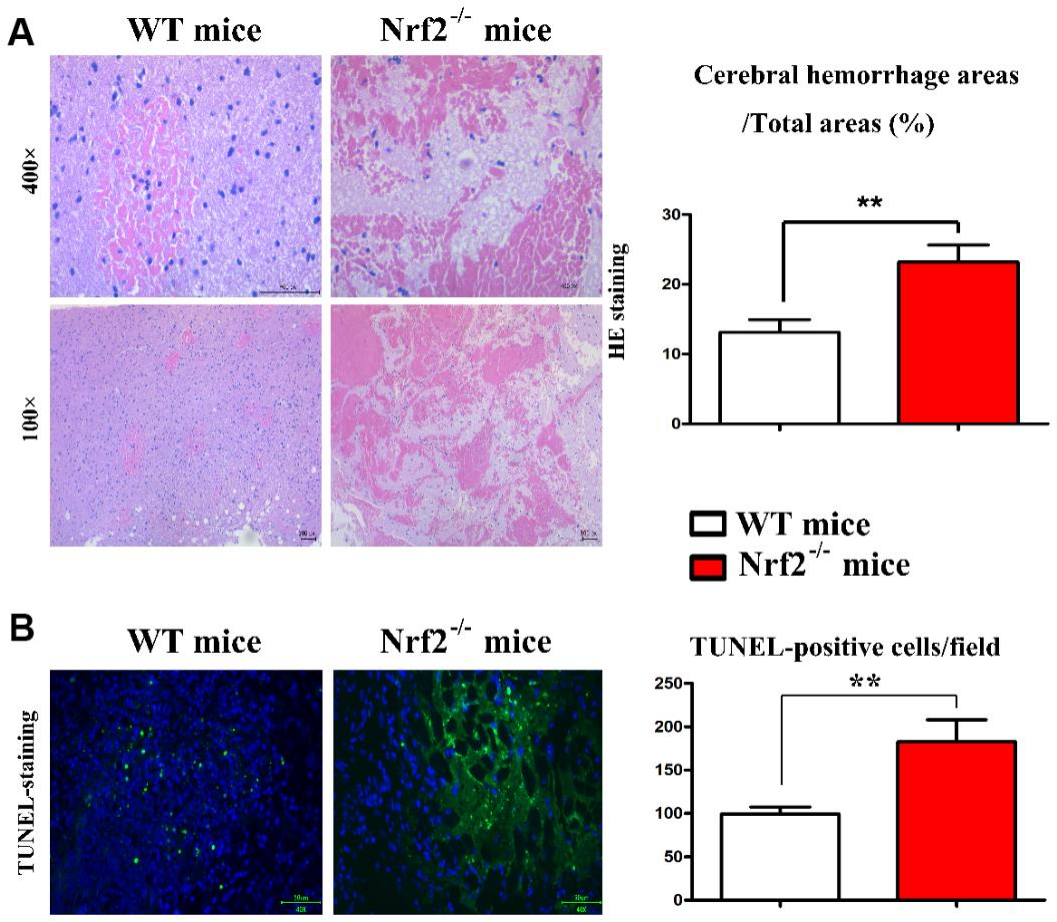
**Histomorphological observation for the brain tissue and TUNEL assay.** (**A**) Histomorphological observation for the brain tissue. (**B**) Effect of Nrf2 on apoptosis of nerve cells in brain injury induced by hydraulic shock.

Under microscope, it was found that the brain tissue structure of all rats in group C was complete, the nerve cells were arranged in order, and the shape was normal. The nucleoli were pale blue and the nuclear membrane was intact, without bleeding, edema and other damage changes. KO mice 24 h after TBI group visible bruises focal point hemorrhage and edema, contusion focal brain tissue around the visible highly porous structure, cell height edema, neuron clearance increases obviously, degeneration of neurons, the cell volume increased obviously, cytoplasm, dye, cell cavity becomes apparent, around with inflammatory cells infiltration and glial cell proliferation. In WT mice, loose tissue structure, mild cell edema, enlarged neuronal space, degeneration of some neurons, and vacuolation in some parts were also observed in the brain tissues around the contusion at 24 h. However, the degree of degeneration and edema in WT mice was significantly less than that in KO mice (Figure 1A).

### Effect of Nrf2 on apoptosis of nerve cells in brain injury induced by hydraulic shock

TUNEL assay was used to detect the apoptotic state of brain tissue, and the TUNEL positive cells were marked as green. There were only a few positive cells in the C group. Compared with C group, the number of TUNEL positive apoptotic cells in the brain tissue around the contusion lesion in TBI group was significantly increased. The apoptosis index of WT mice in TBI group was lower than that in KO mice (*P*<0.05), the difference was statistically significant (*P*<0.05) (Figure 1B).

### Screening of DEGs

**Figure 2:**
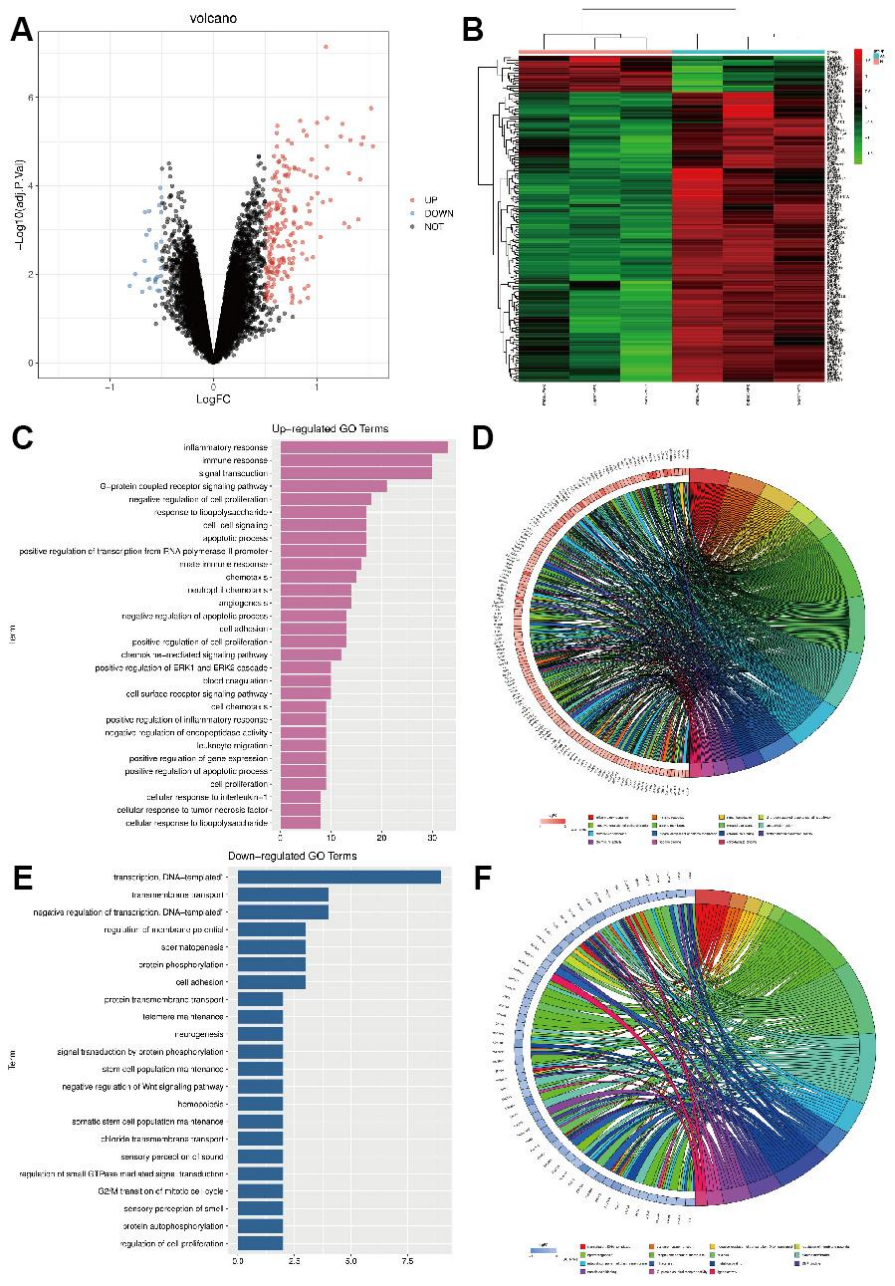
(**A**) GSE24265 differential gene volcano map; (**B**) GSE24265 differential gene cluster analysis heat map; (**C**, **D**) GO enrichment analysis of the up-regulated pathway; (**E**, **F**) GO enrichment analysis of down-regulated pathway.

Data set GSE24265 related to cerebral hemorrhage was downloaded from GEO database. Using R language limma package for data set GSE24265 quantile standardized preprocessing, differences in gene analysis (|logFC|<0.5, *p*-value<0.05), and related differential genes were obtained, including 202 up-regulated genes and 28 down-regulated genes. Volcano maps (Figure 2A) and cluster analysis heat maps (Figure 2B) were drawn for the differential genes.

### Functional enrichment analysis

**Figure 3:**
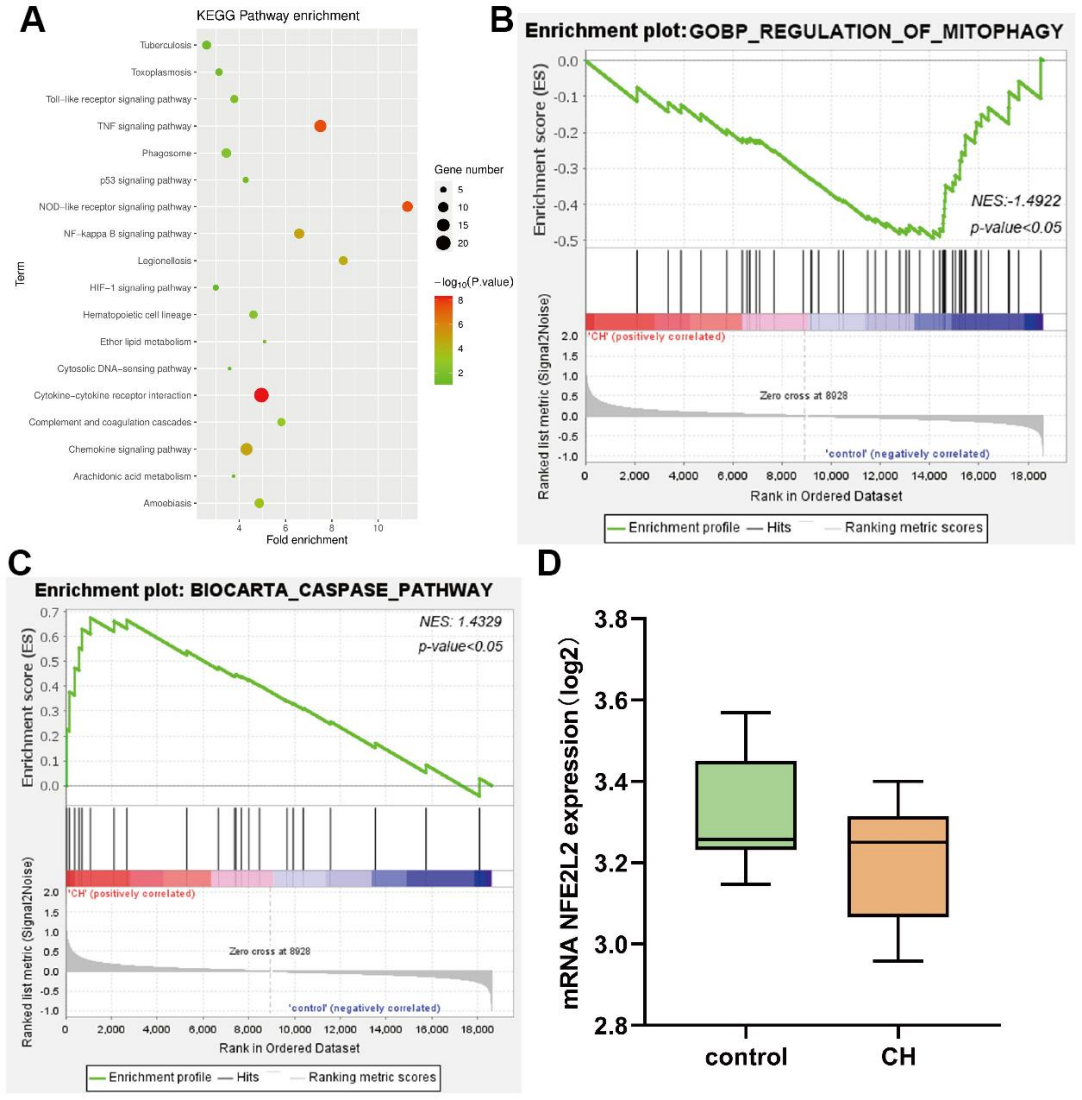
(**A**) KEGG pathway enrichment analysis; (**B**, **C**) GSEA gene enrichment analysis; (**D**) The expression analysis of Nrf2.

GO enrichment analysis was performed on DEGS genes using the online tool DAVID, and the up-regulation pathway map of GO was drawn using R language (Figure 2C, 2D), which indicated that the genes were enriched in inflammatory response, immune response, apoptosis pathway, etc. GO down-regulated pathways (Figure 2E, 2F), including transcription, cell adhesion, etc. Differential genes were used to analyze the KEGG pathway and draw the KEGG pathway map, which indicated that the cytokine-cytokine receptor interaction and other related pathways were enriched (Figure 3A). In addition, GSEA gene enrichment analysis revealed enrichment in mitophagy, CASPASE_PATHWAY and other pathways (Figure 3B, 3C).

### Statistical analysis of target genes

The content of Nrf2 in different groups was analyzed by statistical methods, and it could be seen that the expression of Nrf2 was low in the disease group and high in the control group (Figure 3D).

### Effects of Nrf2 knockout on mitophagy and apoptosis induced by fluid percussion brain injury *in vivo*

**Figure 4:**
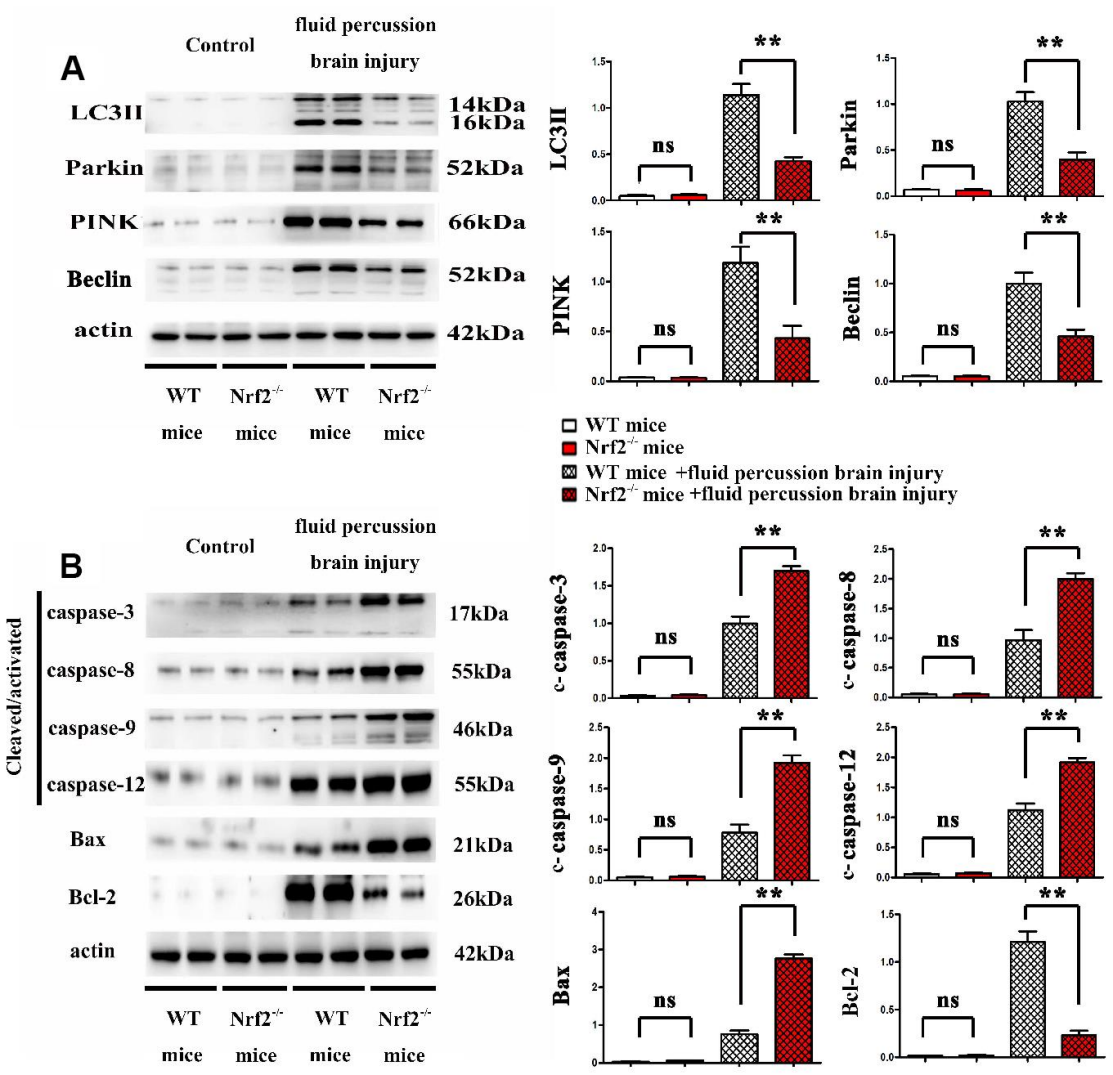
(**A**, **B**) Effects of Nrf2 knockout on mitophagy and apoptosis induced by fluid percussion brain injury *in vivo*.

Whether Nrf2 knockout was able to affect the expression of mitophagy-related proteins and apoptosis-related proteins induced by fluid percussion brain injury *in vivo* was assessed by western blot. Actin was used as a loading control. Bar graphs, representative blots from four independent experiment groups were shown. Representative Western blot images and summarized data showed that Nrf2 knockout decreased the expression of mitophagy-related proteins LC3II, Parkin, Pink and Beclin, while Nrf2 knockout decreased the expression of antiapoptotic protein Bcl-2 and increased the expression of proapoptotic proteins c-caspase-3, c-caspase-8, c-caspase-9, c-caspase-12 and Bax. The data are represented as the mean ± the standard error (SEM). **, *P* < 0.01. The error bars represent the SEM. Statistical analysis was performed using an unpaired, two-tailed Student's t-test (Figure 4).

### Effects of Nrf2 knockout on mitophagy and apoptosis induced by mechanical pressure in glial cells

**Figure 5:**
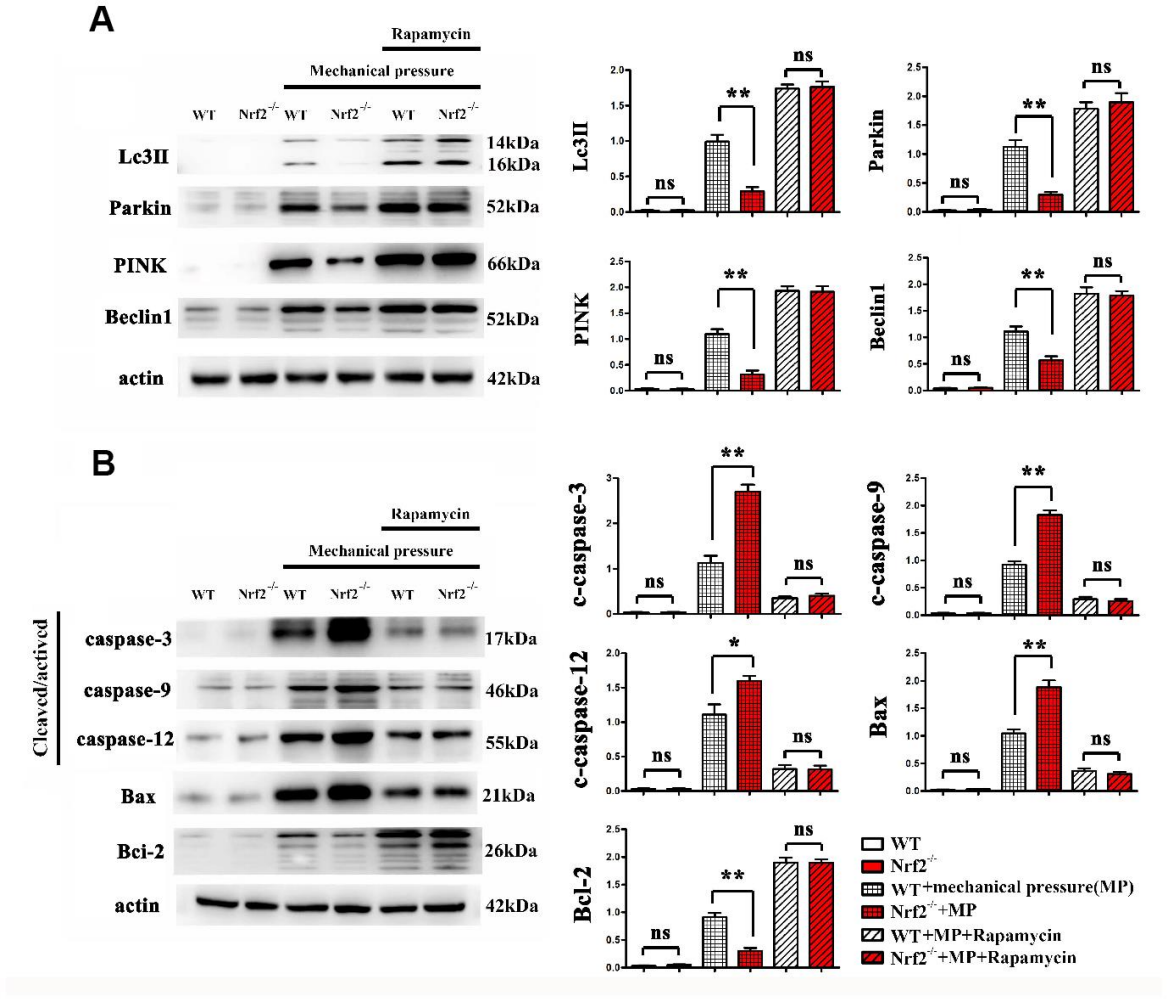
(**A**, **B**) Effects of Nrf2 knockout on mitophagy and apoptosis induced by mechanical pressure in glial cells.

Whether Nrf2 knockout was able to affect the expression of mitophagy-related proteins and apoptosis-related proteins induced by mechanical pressure in glial cells was assessed by western blot. Actin was used as a loading control. Bar graphs, representative blots from four independent experiment groups were shown. Representative Western blot images and summary data showed that Nrf2 knockout decreased the expression of mitochondrial autophagy related proteins Lc3II, Parkin, Pink and Beclin in the mechanically damaged mouse model compared with the control group. Knockout of Nrf2 decreased the expression of anti-apoptotic protein Bcl-2, and increased the expressions of mechanical stress-induced pro-apoptotic proteins c-caspase-3, c-caspase-9, c-caspase-12 and Bax. Rapamycin is an inducer of mitophagy. Results showed that Nrf2 knockout did not affect the expression of mitophagy-related proteins and apoptosis-related proteins induced by mechanical pressure and Rapamycin *in vivo*. The data are represented as the mean ± the standard error (SEM). **, *P* < 0.01. The error bars represent the SEM. Statistical analysis was performed using an unpaired, two-tailed Student's t-test (Figure 5).

## DISCUSSION

The pathophysiological process of craniocerebral injury includes primary injury caused by transient mechanical shock and secondary injury caused by a series of biochemical processes initiated after injury. Secondary brain injury is the focus of clinical intervention, which often induces a large number of reactive oxygen species and free radicals, triggering a series of cascading reactions such as lipid peroxidation, protein oxidation and hydrolysis, and nucleic acid chain break, aggravating cell damage [3, 19]. Studies have shown that the application of NRF2 activator can reduce neuronal death, brain injury volume and neurological function impairment after TBI injury, improve visual and positional memory after TBI injury, reduce neurological dysfunction, and play a neuroprotective role [20]. In our experiment, after gene knockout mice were used to make traumatic brain injury models *in vivo* and *in vitro*, it was found that KO mice and WT mice suffered the same intensity of traumatic brain injury. The damage degree of brain tissue *in vivo* and glial cells *in vitro* of KO mice was significantly stronger than that of WT mice. However, there were no significant changes in all indicators in KO and WT mice in the control group, thus proving that Nrf2 plays an important role in neuroprotective effect after traumatic brain injury, which is consistent with previous literature reports [21, 22].

Nrf2 (nuclear factor erythroid 2. related factor 2), a nuclear transcription factor belonging to the alkaline leucine zipper Cap-nCollar family, is responsible for controlling and coordinating the coding of detoxification enzymes, drug transporter proteins, anti-inflammatory proteins. A group of inducible genes expressed by genes such as antiapoptotic proteins and proteasomes that are expressed in almost all cells. Studies have shown that under normal condition, because of the existence of inhibitors (INrf2) in the cytoplasm, Nrf2 in a constant state of dynamic equilibrium, however when oxidative stress or other external stimuli, Nrf2 from within the cytoplasm to the nucleus, start the related the protein sequence control, reduce the body free electrophilic and reactive oxygen species, increased genetic stability, inhibition of gene mutations, thus play a role in reducing inflammation, reducing oxidation, and inhibiting cell apoptosis [23]. In recent years, Calkins et al. have found that NRT2 pathway is activated after traumatic brain injury and plays a protective role in traumatic brain injury by regulating the activation of glial cells [24]. It plays a protective role in traumatic brain injury [25]. New evidence suggests that NRF2 regulates mitochondrial function and metabolism [26]. However, the protective effect and mechanism of Nrf2 on hydraulic shock brain injury in mice through Pink1-mediated mitophagy have not been reported. Therefore, in order to determine the protective effect and mechanism of Nrf2 on brain injury caused by hydraulic shock in mice, we detected the protein levels of Parkin, Pink, Beclin, LC-3II, Caspase3 and Caspase12. In both *in vitro* and *in vivo* experiments, the protein expressions of Parkin, Pink, Beclin and LC-3II in KO mice were lower than those in WT mice, while the protein expressions of Caspase3 and Caspase12 in TBI mice were higher than those in WT mice. As expected, Nrf2 plays a protective role in nerve cells by enhancing autophagy *in vivo* by enhancing mitochondrial autophagy related proteins after brain injury caused by hydraulic shock. When Nrf2 protein was knocked out, autophagy level decreased, apoptosis increased, and nerve cell damage increased. Mitophagy refers to the process of selective removal of damaged or redundant mitochondria through the autophagy/lysosomal pathway [27]. Autophagy can improve the function of nerve cells by regulating the metabolism of intracellular substances and REDOX balance, and has a neuroprotective effect. Nrf2 is not only the central molecule of the anti-oxidative stress system in the body, but also the transcription factor of a variety of autophagy-related genes, which is cross-regulated with autophagy.

The Pink1/Parkin pathway is believed to be a sensor of mitochondrial quality, regulating the process of mitophagy, and Pink1/Parkin-mediated mitochondrial phagocytosis may prevent cell damage [28]. In this study, the expressions of Parkin and Pink were significantly decreased after brain injury. In addition, the expressions of Parkin and Pink in KO mice were significantly lower than those in WT mice, suggesting that mitophagy controlled by the Pink1/Parkin pathway is involved in the regulation mechanism of hydroshock brain injury, and Nrf2 can promote mitophagy to protect neurons after hydroshock brain injury by regulating Parkin and Pink. At the same time, the results of this experiment showed that 24h after hydraulic shock brain injury, the flow cytometry and TUNEL results showed increased apoptosis, which was consistent with the protein expression trend of Caspase3 and Caspase12. Meanwhile, the water content of brain tissue also showed the same change. These results indicate that the brain injury induced by hydraulic shock can induce apoptosis represented by caspase-3. Caspase-3 is a marker protein in the apoptotic pathway, and the increase of caspase-3 indicates the occurrence of apoptosis [8]. Studies have shown that traumatic brain injury can lead to damage of the gray matter and white matter structure of the brain, especially the oliodendrocytes. After injury, the expressions of Caspase3, tyrosine kinase receptor B (TrkB) and glial fibrous acidic protein are increased, and the expressions of these genes are more obvious in the cerebellum [29]. The apoptosis of oligoendrocytes in the injured area may be related to the increase of caspase-3 [30]. In addition, studies have shown that the expression of Caspase-3 positive cells is significantly increased in neurons of degenerated traumatic brain tissue, and the application of phenyl ethyl caffeinate (CAPE, an anticancer drug) can effectively prevent oxidative reaction induced by trauma [31]. Therefore, taking apoptosis as the breakthrough point of research to prevent the apoptosis of oligodendrocytes caused by trauma and the subsequent demyelinating reaction and conduction disorders can effectively reduce the disability rate and mortality. Caspase12 is the only enzyme in the endoplasmic reticulum of the Caspase family. Under normal conditions, progenosomes exist in the form of progenosomes, which transfer from the cytoplasm to the cell membrane after activation, and are key promoters in the ERS-mediated apoptosis pathway and can only be activated by ERS signals. After activation, Caspase9 can be activated, and then downstream sequences can be activated. Caspase3 is the final executor of cell apoptosis in the Caspase family and plays a key role in cell apoptosis. Activated Caspase3 acts on different substrates, hydrolyzes specific proteins, and eventually leads to irreversible cell death, so it is called “death protein” [32]. Although many important apoptotic proteins have been identified, the understanding of the mechanisms of apoptosis is limited, so the treatment of traumatic brain injury remains a difficult task. Caspase3 and Caspase12 play an important role in the process of cell apoptosis and play an important role in the mechanism of cell apoptosis, while the high expression of Caspase12 is an important reason for the activation of Caspase3 leading to cell apoptosis [33]. Similarly, in the TBI group, the expressions of Caspase3 and Caspase12 in WT mice were significantly higher than those in KO mice, indicating that Nrf2 had an inhibitory effect on cell apoptosis induced by hydraulic shock.

## CONCLUSIONS

In conclusion, Nrf2 plays a protective role by promoting mitophagy to inhibit apoptosis in the process of brain injury caused by hydraulic shock in mice.

## References

[1] Liang Y, Song P, Chen W, Xie X, Luo R, Su J, Zhu Y, Xu J, Liu R, Zhu P, Zhang Y, Huang M. Inhibition of Caspase-1 Ameliorates Ischemia-Associated Blood-Brain Barrier Dysfunction and Integrity by Suppressing Pyroptosis Activation. Front Cell Neurosci. 2021; 14:540669. 10.3389/fncel.2020.540669 33584203 PMC7874210

[2] Do PT, Wu CC, Chiang YH, Hu CJ, Chen KY. Mesenchymal Stem/Stromal Cell Therapy in Blood-Brain Barrier Preservation Following Ischemia: Molecular Mechanisms and Prospects. Int J Mol Sci. 2021; 22:10045. 10.3390/ijms221810045 34576209 PMC8468469

[3] Hernández IH, Villa-González M, Martín G, Soto M, Pérez-Álvarez MJ. Glial Cells as Therapeutic Approaches in Brain Ischemia-Reperfusion Injury. Cells. 2021; 10:1639. 10.3390/cells10071639 34208834 PMC8305833

[4] Ashrafi G, Schwarz TL. The pathways of mitophagy for quality control and clearance of mitochondria. Cell Death Differ. 2013; 20:31–42. 10.1038/cdd.2012.81 22743996 PMC3524633

[5] Abate M, Festa A, Falco M, Lombardi A, Luce A, Grimaldi A, Zappavigna S, Sperlongano P, Irace C, Caraglia M, Misso G. Mitochondria as playmakers of apoptosis, autophagy and senescence. Semin Cell Dev Biol. 2020; 98:139–53. 10.1016/j.semcdb.2019.05.022 31154010

[6] Guidelines for the use and interpretation of assays for monitoring autophagy (3rd edition). Autophagy. 2016; 12:1–222. 10.1080/15548627.2015.1100356 26799652 PMC4835977

[7] Zhao F, Liu C, Fang L, Lu H, Wang J, Gao Y, Gabbianelli R, Min W. Walnut-Derived Peptide Activates PINK1 via the NRF2/KEAP1/HO-1 Pathway, Promotes Mitophagy, and Alleviates Learning and Memory Impairments in a Mice Model. J Agric Food Chem. 2021; 69:2758–72. 10.1021/acs.jafc.0c07546 33591165

[8] Xu B, Zhu L, Chu J, Ma Z, Fu Q, Wei W, Deng X, Ma S. Esculetin improves cognitive impairments induced by transient cerebral ischaemia and reperfusion in mice via regulation of mitochondrial fragmentation and mitophagy. Behav Brain Res. 2019; 372:112007. 10.1016/j.bbr.2019.112007 31238056

[9] Jiang XS, Chen XM, Hua W, He JL, Liu T, Li XJ, Wan JM, Gan H, Du XG. PINK1/Parkin mediated mitophagy ameliorates palmitic acid-induced apoptosis through reducing mitochondrial ROS production in podocytes. Biochem Biophys Res Commun. 2020; 525:954–61. 10.1016/j.bbrc.2020.02.170 32173525

[10] Mattson MP, Gleichmann M, Cheng A. Mitochondria in neuroplasticity and neurological disorders. Neuron. 2008; 60:748–66. 10.1016/j.neuron.2008.10.010 19081372 PMC2692277

[11] Li X, Shi Z, Zhu Y, Shen T, Wang H, Shui G, Loor JJ, Fang Z, Chen M, Wang X, Peng Z, Song Y, Wang Z, et al. Cyanidin-3-O-glucoside improves non-alcoholic fatty liver disease by promoting PINK1-mediated mitophagy in mice. Br J Pharmacol. 2020; 177:3591–607. 10.1111/bph.15083 32343398 PMC7348088

[12] Doblado L, Lueck C, Rey C, Samhan-Arias AK, Prieto I, Stacchiotti A, Monsalve M. Mitophagy in Human Diseases. Int J Mol Sci. 2021; 22:3903. 10.3390/ijms22083903 33918863 PMC8069949

[13] Sun B, Yang S, Li S, Hang C. Melatonin Upregulates Nuclear Factor Erythroid-2 Related Factor 2 (Nrf2) and Mediates Mitophagy to Protect Against Early Brain Injury After Subarachnoid Hemorrhage. Med Sci Monit. 2018; 24:6422–30. 10.12659/MSM.909221 30210141 PMC6149238

[14] Fan RF, Tang KK, Wang ZY, Wang L. Persistent activation of Nrf2 promotes a vicious cycle of oxidative stress and autophagy inhibition in cadmium-induced kidney injury. Toxicology. 2021; 464:152999. 10.1016/j.tox.2021.152999 34695510

[15] Chiang S, Huang MLH, Park KC, Richardson DR. Antioxidant defense mechanisms and its dysfunctional regulation in the mitochondrial disease, Friedreich’s ataxia. Free Radic Biol Med. 2020; 159:177–88. 10.1016/j.freeradbiomed.2020.07.019 32739593

[16] Pan H, Guan D, Liu X, Li J, Wang L, Wu J, Zhou J, Zhang W, Ren R, Zhang W, Li Y, Yang J, Hao Y, et al. SIRT6 safeguards human mesenchymal stem cells from oxidative stress by coactivating NRF2. Cell Res. 2016; 26:190–205. 10.1038/cr.2016.4 26768768 PMC4746611

[17] Brandes MS, Zweig JA, Tang A, Gray NE. NRF2 Activation Ameliorates Oxidative Stress and Improves Mitochondrial Function and Synaptic Plasticity, and in A53T α-Synuclein Hippocampal Neurons. Antioxidants (Basel). 2021; 11:26. 10.3390/antiox11010026 35052530 PMC8772776

[18] Balentine JD, Greene WB, Bornstein M. *In vitro* spinal cord trauma. Lab Invest. 1988; 58:93–9. 3336206

[19] Shaheryar ZA, Khan MA, Adnan CS, Zaidi AA, Hänggi D, Muhammad S. Neuroinflammatory Triangle Presenting Novel Pharmacological Targets for Ischemic Brain Injury. Front Immunol. 2021; 12:748663. 10.3389/fimmu.2021.748663 34691061 PMC8529160

[20] Saykally JN, Rachmany L, Hatic H, Shaer A, Rubovitch V, Pick CG, Citron BA. The nuclear factor erythroid 2-like 2 activator, tert-butylhydroquinone, improves cognitive performance in mice after mild traumatic brain injury. Neuroscience. 2012; 223:305–14. 10.1016/j.neuroscience.2012.07.070 22890082 PMC9150436

[21] Song J, Du G, Wu H, Gao X, Yang Z, Liu B, Cui S. Protective effects of quercetin on traumatic brain injury induced inflammation and oxidative stress in cortex through activating Nrf2/HO-1 pathway. Restor Neurol Neurosci. 2021; 39:73–84. 10.3233/RNN-201119 33612499

[22] Zhang XS, Lu Y, Li W, Tao T, Peng L, Wang WH, Gao S, Liu C, Zhuang Z, Xia DY, Hang CH, Li W. Astaxanthin ameliorates oxidative stress and neuronal apoptosis via SIRT1/NRF2/Prx2/ASK1/p38 after traumatic brain injury in mice. Br J Pharmacol. 2021; 178:1114–32. 10.1111/bph.15346 33326114

[23] Chang SH, Lee JS, Yun UJ, Park KW. A Role of Stress Sensor Nrf2 in Stimulating Thermogenesis and Energy Expenditure. Biomedicines. 2021; 9:1196. 10.3390/biomedicines9091196 34572382 PMC8472024

[24] Calkins MJ, Vargas MR, Johnson DA, Johnson JA. Astrocyte-specific overexpression of Nrf2 protects striatal neurons from mitochondrial complex II inhibition. Toxicol Sci. 2010; 115:557–68. 10.1093/toxsci/kfq072 20211941 PMC2871759

[25] Dai W, Wang H, Fang J, Zhu Y, Zhou J, Wang X, Zhou Y, Zhou M. Curcumin provides neuroprotection in model of traumatic brain injury via the Nrf2-ARE signaling pathway. Brain Res Bull. 2018; 140:65–71. 10.1016/j.brainresbull.2018.03.020 29626606

[26] Dinkova-Kostova AT, Abramov AY. The emerging role of Nrf2 in mitochondrial function. Free Radic Biol Med. 2015; 88:179–88. 10.1016/j.freeradbiomed.2015.04.036 25975984 PMC4726722

[27] Bingol B, Sheng M. Mechanisms of mitophagy: PINK1, Parkin, USP30 and beyond. Free Radic Biol Med. 2016; 100:210–22. 10.1016/j.freeradbiomed.2016.04.015 27094585

[28] Liu Y, Yan J, Sun C, Li G, Li S, Zhang L, Di C, Gan L, Wang Y, Zhou R, Si J, Zhang H. Ameliorating mitochondrial dysfunction restores carbon ion-induced cognitive deficits via co-activation of NRF2 and PINK1 signaling pathway. Redox Biol. 2018; 17:143–57. 10.1016/j.redox.2018.04.012 29689442 PMC6006734

[29] Staffa K, Ondruschka B, Franke H, Dreßler J. Cerebellar gene expression following human traumatic brain injury. J Neurotrauma. 2012; 29:2716–21. 10.1089/neu.2011.2246 23030803

[30] Lotocki G, de Rivero Vaccari JP, Alonso O, Molano JS, Nixon R, Safavi P, Dietrich WD, Bramlett HM. Oligodendrocyte vulnerability following traumatic brain injury in rats. Neurosci Lett. 2011; 499:143–8. 10.1016/j.neulet.2011.05.056 21669255 PMC3523350

[31] Kerman M, Kanter M, Coşkun KK, Erboga M, Gurel A. Neuroprotective effects of caffeic acid phenethyl ester on experimental traumatic brain injury in rats. J Mol Histol. 2012; 43:49–57. 10.1007/s10735-011-9376-9 22124729

[32] Dhani S, Zhao Y, Zhivotovsky B. A long way to go: caspase inhibitors in clinical use. Cell Death Dis. 2021; 12:949. 10.1038/s41419-021-04240-3 34654807 PMC8519909

[33] Sun GZ, Gao FF, Zhao ZM, Sun H, Xu W, Wu LW, He YC. Endoplasmic reticulum stress-induced apoptosis in the penumbra aggravates secondary damage in rats with traumatic brain injury. Neural Regen Res. 2016; 11:1260–6. 10.4103/1673-5374.189190 27651773 PMC5020824

